# The Prevalence of Simultaneously Ordering Amylase and Lipase for Diagnosing Pancreatitis

**DOI:** 10.1155/2023/3988278

**Published:** 2023-09-27

**Authors:** Bader Alyahya, Abdulaziz Alalshaikh, Abdulaziz Altaweel, Gadah Alsaleh, Abdullah Alsaeed, Haneen Somily, Taif Alotaibi, Mohammed Alaqeel, Abdulaziz Al Mehlisi, Fahad Abuguyan, Fawaz Altuwaijri, Zohair Al Aseri

**Affiliations:** ^1^College of Medicine, King Saud University, Riyadh, Saudi Arabia; ^2^College of Medicine, Riyadh Hospital Dar Al Uloom University, Riyadh, Saudi Arabia

## Abstract

**Background:**

The simultaneous measurement of serum amylase and lipase levels in the diagnosis of pancreatitis was deemed unnecessary in several studies. We aim at evaluating the prevalence of the simultaneous co-ordering of serum amylase and lipase.

**Methods:**

This retrospective chart review was conducted at King Saud University Medical City in Riyadh, Saudi Arabia, between January 2021 and January 2022. We examined requests for serum amylase or serum lipase levels that had been sought for suspected pancreatitis within the electronic health system (EHS).

**Results:**

A total of 9,617 requests for serum amylase and serum lipase levels for 5,536 patients were made in a year; 6,873 (71.5%) were made for serum lipase alone; 1,672 (17.4%) were made for co-ordered serum lipase and amylase; 322 (3.3%) were made for amylase alone; and 750 (7.8%) were made for repeated amylase testing. Four hundred and thirteen tests (4.3%) yielded a diagnosis of pancreatitis. The estimated cost reduction when serum amylase was removed if serum lipase was co-ordered was 108,680 SAR (approximately US$28,960).

**Conclusion:**

Serum amylase and lipase were co-ordered for about 17.4% of pancreatitis diagnostic tests, all of which were unnecessary. Eliminating serum amylase testing for any patient who receives a test of their lipase levels would exert a significant impact on institutional costs and savings.

## 1. Introduction

When pancreatic enzymes cause autodigestion, an inflammatory cascade ensues that precipitates the loss of pancreatic tissue, resulting in an inflammatory condition called pancreatitis. This illness frequently results in an emergency department visit and hospitalization. The mortality rate resulting from acute pancreatitis can be as high as 30% in the most severe instances; typically, it ranges from 4% to 10% [1].

The exocrine pancreatic enzymes amylase and lipase are used in the evaluation process used to identify pancreatitis. Research has suggested that serum lipase levels are a more sensitive and specific indicator of pancreatitis [1, 2]. The diagnostic consistency of serum lipase to diagnose pancreatitis was found to be better compared to serum amylase [3, 4].

Although both can be utilized, according to guidelines from the American College of Gastroenterology (ACG), serum lipase testing is preferred over serum amylase testing for the diagnosis of pancreatitis, as serum amylase levels alone are not reliable for diagnosing pancreatitis compared to serum lipase [5]. Similarly, the Canadian Practice Guidelines (CPG) recommend that individuals suspected of having acute pancreatitis should undergo a serum lipase test alone, with a threshold of a threefold increase in lipase levels [6]. In addition, numerous studies have demonstrated significant cost reductions after the measurement of serum amylase levels was eliminated from the order list. Costs declined by over 77%, with annual cost savings ranging from $135,000 to $350,000 USD [7, 8].

Ordering both tests concurrently has demonstrated no diagnostic utility; rather, it is associated with increased costs [5–8]. However, the previous literature indicates that the practice of ordering both tests is prevalent, with 91% of patients in large academic centers in the United States having both ordered during testing for pancreatitis, as well as in Canada [9, 10]. Considering the lack of local data regarding the practice of diagnosing pancreatitis, we sought to understand the frequency of this practice and conduct a cost analysis regarding unnecessary serum amylase requests.

## 2. Methods

This single-center retrospective chart review was conducted at King Saud University Medical City (KSUMC) in Riyadh, Saudi Arabia, between January 2021 and January 2022. We searched the electronic health system (EHS) for patients aged 14 or older whose serum amylase or serum lipase levels had been sought for the diagnosis of pancreatitis. The study included all adult patients who were suspected of having pancreatitis. Patients with a confirmed diagnosis of a salivary gland stone or tumor were excluded. We reviewed the EHS for the final clinical diagnoses to assess whether the elevated biomarkers were due to pancreatitis or an alternative diagnosis.

Demographic information included the patient's age, gender, the etiology of the pancreatitis, and their levels of serum amylase and serum lipase. To calculate the costs for each amylase and lipase test, we collected the current cost of each test from various government and private laboratories in Riyadh, Saudi Arabia. The Statistical Package for Social Sciences (SPSS) version 26.0 was used to analyze the data (IBM-SPSS, Armonk, New York, USA). For categorical variables, the results were presented as numbers and percentages, and for continuous variables, they were presented as means and standard deviations. Statistical significance was defined as corresponding to a *p* value of <0.05. The Institutional Review Board (IRB) of the Deanship of Scientific Research within the College of Medicine at King Saud University in Riyadh, Saudi Arabia, granted ethical approval to conduct the study (Project number E-22-6787).

## 3. Results

A total of 9,617 requests for serum amylase and serum lipase levels for 5,536 patients were made during the study period. There were 2,249 (40.6%) males and 3,287 (59.4%) females. The mean age during the visits was 43.4 ± 19.6 years (minimum 14, maximum 109 years). Half (*n* = 4,855, 50.5%) of the requests were made in the emergency department.  Table 1 indicates the basic characteristics of the study population.

**Figure 1:**
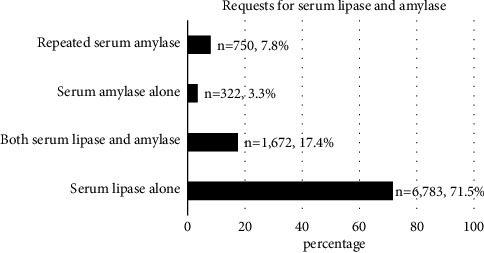
Serum lipase and serum amylase requests.

Of the total requests made, 6,873 (71.4%) were requests for serum lipase, 1,672 (17.4%) were requests for both serum lipase and amylase, 322 (3.3%) were requests for serum amylase alone, and 750 (7.8%) were repeated requests for serum amylase (Figure 1). Requests for both serum amylase and lipase were made significantly more frequently among female patients (62.3% versus 37.7%, *p* = 0.010). No significant differences were evident in the proportion of requests for serum amylase only and for serum lipase only according to gender (3.8% versus 4.3%, *p* = 0.352).

**Figure 2:**
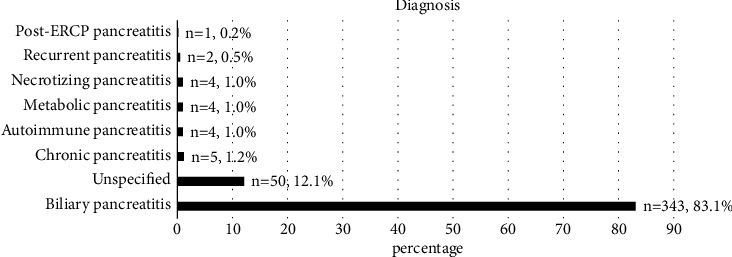
List of the diagnoses yielded by 413 tests of patients with confirmed pancreatitis.

Figure 2 illustrates the underlying etiology of pancreatitis. Of all the tests performed, 413 (4.3%) yielded a diagnosis of pancreatitis, 343 (83.1%) indicated biliary pancreatitis, 50 (12.1%) indicated an unspecified cause, and 20 (4.8%) indicated other causes. Furthermore, the diagnoses were confirmed via serum lipase in 385 (93.2%) cases, and 28 (6.8%) involved serum amylase.

For the tests associated with a confirmed diagnosis of pancreatitis, the diagnostic threshold used for serum amylase is 117.5 U/L (95% CI = 0.753–0.827, *p* < 0.001). For the serum lipase, the diagnostic threshold used was 219 U/L (95% CI = 0.968–0.983, *p* < 0.001).

The current average cost of one lipase test in Riyadh is 150 SR, whereas the current average cost for an amylase test is 130 SR. In 1,672 tests in which both serum amylase and lipase were used, the estimated cost reduction if serum amylase was removed from the request was 108,680 SAR (1,672 tests/2, equaling 836 serum amylase tests), which is equivalent to approximately US$28,960.

## 4. Discussion

This study's objectives were to determine the prevalence of simultaneous requests of serum amylase and lipase for suspected pancreatitis and to perform a cost analysis regarding serum amylase requests that may not be required to diagnose pancreatitis.

In this study, we found that simultaneous requests for serum lipase and amylase tests accounted for 17.4% of all requests; although this is lower than the percentage reported in recent literature (Ritter 2020), according to which 86% of amylase tests were ordered with lipase, our findings still demonstrated an unnecessary cost and burden on laboratory services [11]. Serum amylase tests only accounted for 3.3% of all requests. In actuality, the diagnosis of pancreatitis was only supported by 6.8% of the serum amylase orders. The International Association of Pancreatology, the United Kingdom Working Group in 2005, the Japanese guidelines, the Italian Society of Clinical Biochemistry and Clinical Molecular Biology, the American College of Gastroenterology, and the Canadian Practice Guideline have all recommended which laboratory test should be used for the diagnosis of acute pancreatitis, expressing a preference for serum lipase levels [5, 12–17]. Serum lipase's longer half-life is one of the factors that contributed to this preference [9]. Physicians' requests for both tests in the diagnosis of pancreatitis can be attributed to several reasons. Many practitioners requested both amylase and lipase simultaneously because they believed it would improve the diagnostic precision of both tests in patients presenting with abdominal pain [18, 19]. Other possible explanations for why many practitioners request both tests simultaneously relate to security, trust, and the prevention of medical malpractice claims [20].

In our study, 7.8% of serum amylase tests were repeated, and 3.3% were requested for serum amylase alone, which was lower than the rate of 12.4% reported in other studies. Consequently, only 3.6% of the tests were diagnostic for acute pancreatitis [21]. This is in accordance with many investigations that have found that simultaneous requests for both serum amylase and lipase are unnecessary, since serum lipase alone has displayed better sensitivity and specificity compared to serum amylase [7, 8, 18–22]. Serum lipase has been demonstrated to have a higher diagnostic sensitivity than serum amylase [23]. Several retrospective and cohort studies have proven that simultaneous tests using lipase and amylase are unnecessary and some suggested that serum lipase alone is enough to diagnose pancreatitis [23–25]. Studies have found that serum lipase has a diagnostic accuracy rate of up to 91.8%; conversely, serum amylase has an accuracy rate of only 40.3% [26, 27].

The price of amylase and lipase tests was another subject of this investigation. We calculated a cost reduction of $28,960 when serum amylase was not co-ordered with serum lipase simultaneously. In addition, reducing unnecessary testing would reduce the burden on laboratory staff. Unnecessary testing may also lead to false positive results, which may further guide physicians to order confirmatory tests, such as ultrasounds or computed tomography scans, particularly considering other causes of elevated serum alongside abdominal pain, such as perforated peptic ulcers, acute cholecystitis, and bowel obstruction [28]. Numerous studies have demonstrated that health care systems would benefit in terms of cost savings from using serum lipase testing alone to identify acute pancreatitis. One study found that removing the amylase test from the order list reduced the amount of co-ordering by 77%, saving $135,000 US annually [7]. Aside from the additional imaging and extended hospitalization, serum amylase testing resulted in needless annual expenditures amounting to around $35,000, resulting in significantly higher costs [26, 29]. In other studies, fewer orders for serum amylase were associated with predicted net cost savings of $44,999 annually and patient costs of as much as $135,000 annually [30, 31]. In addition, one study found that ordering amylase and lipase together frequently yielded contradictory results, which could result in an inaccurate diagnosis or more unnecessary testing when compared to ordering lipase alone. Reducing the co-ordering of lipase and amylase may eliminate unnecessary perplexing results and improve the quality of the treatment provided [8].

This study is limited by the fact that it was conducted at a single institution, which may limit the generalizability of the findings to other institutions and other health care systems; nonetheless, the previous literature supports our findings. The retrospective design of the study is another limitation. However, we were able to demonstrate the concurrent unnecessary usage of amylase and lipase testing to identify acute pancreatitis, as well as the additional costs for the healthcare system.

## 5. Conclusion

In our facility, the co-ordering of serum amylase in conjunction with serum lipase accounted for about 17.4% of pancreatitis diagnostic tests, all of which were unnecessary. Serum amylase has a low utility as suggested in previous several studies in terms of the diagnosis of pancreatitis. Unnecessary serum amylase testing for any patient receiving a test of their lipase serum levels would exert a significant impact on institutions' costs and savings. Future research should assess the effects of decreased co-ordering on healthcare quality and determine whether additional instances of unnecessary diagnostic testing may be prevented using a similar strategy.

## Figures and Tables

**Table 1:** Basic characteristics of the study.

	*N* (%)	Mean ± SD
Age in years		43.4 ± 19.6 (14 to 109 years old)
Gender		
Male	2,249 (40.6%)	
Female	3,287 (59.4%)	
Place of request		
Emergency department	4,855 (50.5%)	
In-patient	4,465 (46.4%)	
Outpatient	233 (2.4%)	
Preadmission	34 (0.4%)	
Telehealth	22 (0.2%)	
Day care	8 (0.1%)	

## Data Availability

The data are available upon reasonable request from the corresponding author.
